# The comparative pathology workbench: An update

**DOI:** 10.1016/j.jpi.2025.100523

**Published:** 2025-10-21

**Authors:** Michael N. Wicks, Michael Glinka, Bill Hill, Derek Houghton, Bernard Haggarty, Jorge Del-Pozo, Ingrid Ferreira, Florian Jaeckle, David Adams, Shahida Din, Irene Papatheodorou, Kathryn Kirkwood, Albert Burger, Richard A. Baldock, Mark J. Arends

**Affiliations:** a Edinburgh Pathology & Centre for Comparative Pathology, Institute of Genetics & Cancer, University of Edinburgh, Crewe Road, Edinburgh EH4 2XR, UK; b Department of Computer Science, School of Mathematical and Computer Sciences, Heriot-Watt University, Edinburgh, UK; c Experimental Cancer Genetics, Wellcome Sanger Institute, Hinxton, Cambridge, UK; d Edinburgh IBD Unit, Western General Hospital, NHS Lothian, Edinburgh, UK; e Earlham Institute, Norwich Research Park, Norwich NR4 7UZ, UK; f The Royal (Dick) School of Veterinary Studies, The University of Edinburgh, Easter Bush Campus, Bush Farm Road, Edinburgh EH25 9RG, UK; g Department of Pathology, University of Cambridge, Cambridge, UK

**Keywords:** Image visualization, Shared workspace, Image spreadsheet, Visual comparison, Visual analytics, Embedded discussion, Comparative pathology

## Abstract

The Comparative Pathology Workbench (CPW) is a web-browser-based visual analytics platform providing shared access to an interactive “spreadsheet” style presentation of image data and associated analysis data. The software was developed to enable pathologists and other clinical and research users to compare histopathological images of diseased and/or normal tissues between different samples of the same or different patients/species. The CPW provides a grid layout of cells in rows and columns so that images that correspond to matching data can be organized in the form of an image-enabled “spreadsheet”. An individual workbench or bench can be shared with other users with read-only or full edit access as required. In addition, each bench cell or the whole bench itself has an associated discussion thread to allow collaborative analysis and consensual interpretation of the data. Here, we present the updated system based on 2 years of active use in the field that generated constructive feedback. The updates deliver new capabilities, including automated importation of entire image collections, sorting image collections, long running tasks, public benches, uploading miscellaneous image types, refining search facilities, enabling use of tags, and improving efficiency, speed, and user-friendliness.

## Introduction

The Comparative Pathology Workbench (CPW) is a web-browser-based visual analytics platform providing shared access to an interactive workbench (or bench) that is a “spreadsheet” style presentation of images and associated analysis data.^6^ It allows a range of medical professionals, including pathologists, and scientists to share and compare images such as histopathological, radiological and spatial transcriptomics images of diseased organs and tissues, for inflammatory and immune disorders, cardiovascular diseases, tumors, and many other conditions. Each displayed image links out to the primary image source with the associated additional analysis and query tools. A key capability of the CPW is a mechanism to capture discussion between users as a thread allowing many users to contribute to the analysis of the data in a structured way. In research mode, the individual workbenches can be shared with selected users and when required can be made public, e.g., as a co-publication of the data associated with a manuscript publication.

**Fig. 1:**
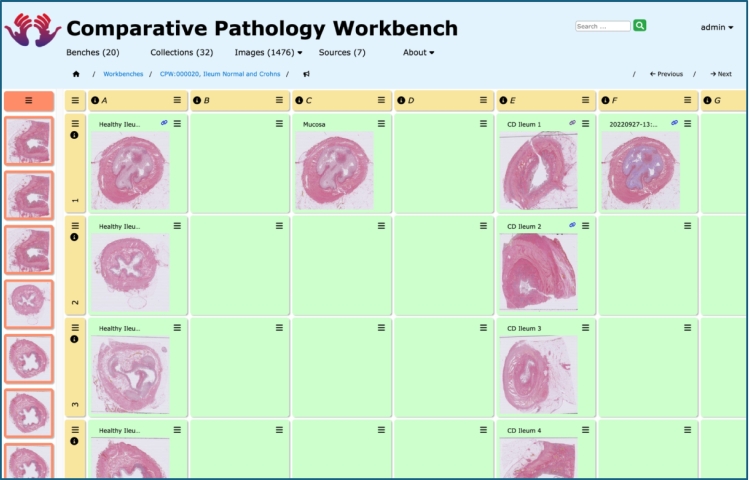
A bench in the Comparative Pathology Workbench system. This shows a typical bench within the CPW system, with the tabular arrangement of rows and columns of cells, and cells populated with images from various sources. The images in the first column show healthy ileum samples, with Crohn's diseased examples in the fifth column. QuPath (open source software for bioimage analysis, digital pathology, and whole slide image analysis) has been used to identify particular biological features, with the relationship between images stored as a link between two images as represented by the “chain” icon.

The CPW offers users a novel approach to comparing images. Previously, pathologists used serial viewing of glass slide sections down a microscope, forcing pathologists to retain the diagnostic features in memory, while comparing a large series of cases, or to print series of photomicrographs at fixed magnifications and to compare these static images. Instead, the CPW allows the pathologist to catalog histopathological section data in workbenches or benches of images, where images are collated, providing a direct means of comparison. The images in a bench are provided “spreadsheet” style in cells which are arranged in rows and columns (see Fig. 1).

An individual bench can be shared with other users with read-only or full edit access as required. In addition, each bench cell containing an image reference, as well as the whole bench itself, has an associated discussion thread to allow collaborative image interpretation and analysis, promoting development of a consensus in interpretation of the data. Image data are typically hosted by other resource applications such as an OMERO server^4^ or remote image archives such as the IDR^3^^,^^7^ and the Cancer Digital Slide Archive.^8^ In addition, miscellaneous images can be stored within the CPW itself and the CPW has been developed to allow integration of image analysis outputs from systems such as QuPath^9^ or ImageJ.^10^ An OMERO server is an image repository system, in contrast to the CPW which leverages such systems by providing a layer of functionality to the user, allowing contextualization and discussion within a specific research domain or project, without requiring the reinvention of an image repository. However, both OMERO and the CPW are web-based systems with all the accessibility benefits that such systems provide. In contrast to the CPW, QuPath is a desktop-only application specifically designed for quantitative image analysis and has no means to share or discuss these analyses via a web-based “back-end” application. Thus, the CPW provides a mechanism to display original images together with their analysis results with processed images generated by QuPath, ImageJ, or similar applications, in a single web-based location, offering CPW users the opportunity to contribute to a discussion thread within the CPW system itself, enhancing interactions between team members involved in the research.

The publication of the original version of the CPW (version 1)^6^^,^^11^^,^^12^ included contributions from three “real-world” exemplars^6^^,^^13^^,^^14^ illustrating the benefits and usage of the system. We have further developed the CPW application in response to feedback comments and suggestions from these groups and other collaborators. We have also extended the available functionality, significantly improved the existing capabilities, improved the computational efficiency and extended the range of sources that can be displayed on the bench.

In addition to providing an update on the CPW to version 2, we describe two new exemplars, one with Crohn's disease related data and the second includes veterinary pathology data used for teaching.

## Materials and methods

The CPW has been developed using open-access tools to ensure license-free distribution with the front-end visualization delivered via a web application written in Python,^15^ using the Django^16^ web development framework, incorporating HTML, CSS, and JavaScript, to provide the image display, user-interface components, and a RESTful API, as well as user and permissions management. The backend SQL database is implemented using PostgreSQL^17^ (version: 11) and the discussion threads and management provided use a WordPress^18^ instance. The overall architecture is described in Wicks et al.^6^

Feedback was sought from existing users and through analysis from monitoring CPW usage we identified a series of requirements and extensions to be implemented. These are:

1. More efficient image import for large image sets. In version 1, image import was incremental and manual, importing one image at a time. For users, this was hugely inefficient and time-consuming for large image sets.
2. Ability for users to sort images within an image collection before importing the whole collection in a specific order within a bench.
3. Import of images from both an image source into a collection, and from a collection into a bench, took considerable processing time causing problems with responsiveness and occasionally an apparently “dead” interface with no feedback.
4. Users required miscellaneous images, including graphs, charts, and diagrams, to be handled by the CPW, not from traditional image handling services like OMERO, but instead generated by desktop and other applications such as Microsoft Excel, QuPath, or ImageJ.
5. Users required the ability to share benches with their colleagues without them having to obtain credentials to access the CPW hosting the bench.
6. Users found the existing search facilities too complicated to use, with query of large collections of images unwieldly, despite being required by the user, without any means to subset collections.
7. Finally, various technical enhancements to the CPW were required to improve system efficiency, speed, and ease of use.

Here, we discuss the technical implementation of these requirements and their appearances to the user are discussed in the results section.

### Multi-image import and cell labeling

The request from users was to enable the import of an entire collection of images directly to a specified location in the bench either as a row (extending the number of columns rightwards) or a column (extending the number of rows downwards). The images imported are all those in the current collection as these are shown on the bench page, at the left-hand side of the screen. With this design, no special processing was required, other than ensuring that the resulting bench contained sufficient rows or columns of cells to accommodate the entire collection. The one additional constraint is that no cell will be over-written if it already houses data, these cells are shifted as required to make space for the collection import. As this import process could now result in the bench dimensions changing significantly, cell labeling had to be improved, therefore cell labeling information (e.g., in the menu) needed to be modified to indicate the cell coordinates, matching the header cell column *letter* and row *number,* to maintain consistency with familiar Microsoft Excel spreadsheet cell naming styles.

### Image sorting within collections

A sorting ability was implemented for collections to allow users to pre-sort images in a specific order within a collection. This was done to allow users to automatically populate benches with sorted images that contain similar properties, e.g., images coming from the same source, or same sample. Previously, images were sorted within a collection in the order in which they were added to that collection. With the addition of new functionality to add entire collections into a bench in a single operation, this default ordering would not increase the efficiency of the system if the user was then forced to spend time rearranging a set of imported images in bench cells. Therefore, a mechanism to sort collections by users before importation into a bench was required.

### Long-running tasks

The creation of large image collections and the population of benches with an entire collection of images often caused the CPW to timeout, and fail to complete the task, often leaving the user with a blank screen reporting “500 Server Error”, which is clearly unacceptable. The issue was that long-running processes blocked the web server until the task had completed with the program failing to respond to the user. The solution we have adopted is to use a task-scheduling system in combination with a message broker (to execute the tasks), such that these long-running processes do not affect the web server and in principle can also be executed on a different server. Control is returned to the user to continue work but with a lock set on the affected bench until the task has been completed, to avoid the possibility that the user might submit a conflicting request.

We have used the open-source Python package Celery^19^ (version 5.4.0) for task scheduling and for the message broker we used Redis^20^ (version 7.2.6). This allows a very simple setup with the scheduler communicating with the message broker via a URL. This software architecture has proved to be reliable and maintenance free, after initial setup.

Before a background task is initiated, any bench or collection impacted is marked as “locked”; once the background task has been completed, the bench or collection is then marked “unlocked”, this being the very last activity of the background task. The CPW is not a transaction intensive system, so this simple level of locking is sufficient, particularly as the locking of a bench also locks all the cells of that bench.

### Public benches

In CPW version 1, only registered users could access and create benches which could only be shared with other registered users. This also applied to the discussion threads, that were associated with specific cells, or benches. This level of control is critical to allow collaboration between users with private and unpublished data. However, a requirement emerged that users wanted to publish their work and in doing so, required the benches and images to be accessible in such a way that would allow non-registered users to view the data. In version 2, therefore, we extend the bench access to include “Public” status to enable access to users without registration and login credentials.

To make full access possible, the user will also need to make any images used open access and without registration. In the context of OMERO, this is possible by defining a “Public” user and allowing that user permission to see the required images.

### Miscellaneous images

The CPW can present references to images in bench cells that are held on various types of image sources, including OMERO servers, WordPress Blogging servers, and the EBI SCEA^2^. However, users may also want to include in their benches, images that are not held this way, such as charts from desktop applications like the Microsoft Office suite of programs or other analysis tools such as QuPath or ImageJ. CPW version 2, therefore, includes a mechanism to import small images with matching metadata, description, and an URL identifying the image source.

### Improved searching

With the ability to bring in very large image collections, it became clear that the means of searching for images needed to be improved in two ways to complement the existing search via parameter selection. First, a search based on a simple text string to bring back “top” hits within each of the image, collection, and bench categories, was required to find images without knowing the specific metadata values. Second, users required the capability to rapidly subset large image collections using the search mechanism. CPW version 2, therefore, includes a simple text search across all categories linked to a new search page and to the global search available in the CPW page banner.

To enable more useful sub-setting of large image sets, we have introduced the option of image “Tags”, which are short text strings that can be assigned to an image as one of multiple tags. Once entered, the tag strings are managed in the database to allow dynamic prompting of tag values ensuring consistent re-use. Typing in a new text string will result in a new tag definition which can then be used for other images. The Advanced Image Search mechanism has been extended to allow query via tags, resulting in rapid identification of the required subset of tagged images. This option has also been made available for the bench image set allowing the subset of images to be made conveniently available for populating the bench.

### Efficiency and user experience improvements

Rendering images within web-based software applications quickly becomes an issue, when many high-resolution images are included in a web page, and the CPW is no different. Benches can easily contain references to many tens of images, possibly even hundreds or thousands, hosted on many external image sources, so generating thumbnail representations in a bench can take a long time and result in the very slow rendering of the web page containing the bench. A mechanism to generate and cache locally thumbnail representations of externally referenced images was required, as was a secure means to render them in the CPW web pages, to avoid them becoming accessible without login credentials. Finally, continual efforts are required during system development to upgrade the system environment, in terms of required supporting software library packages and software systems, to improve the user-friendliness as well as the speed and efficiency of operating the CPW.

### Task performance comparison

In order to quantify and visualize the improvements provided by the automatic image uploading population system, we timed the population of a single bench with a specific number of cells. To estimate the time taken to populate a single cell manually, we created a single collection containing 10 images and started a timer once the first image had been moved to a cell in a bench, stopping the timer once the tenth image populated a cell. This allowed the time for each cell image population to be extracted from the system logs, resulting in a calculated time of 6.55 s to populate a cell manually with an image. For collections containing 1, 10, 50, 100, and 500 images, this manual estimate of image population time, could be extrapolated to 6.55, 65.50, 327.50, 655, and 3275 s, respectively. Switching to the recording of the time it takes to populate a bench automatically using a background task, collections containing 1, 10, 50, 100, and 500 images were created, along with a separate bench for each collection. Each bench created contained sufficient cells in a column to accommodate all the images from the collection. This dimension allocation was done to remove any automatic bench resizing processing from the overall recorded task time, producing an update time solely for the cell image population, and nothing else. For collections containing 1, 10, 50, 100, and 500 images, this automatic task update produced the following times of 8, 15.67, 37, 43, and 210 s, respectively.

### Statistical analysis

Welch's *t*-test for summary statistics was utilized to determine significant differences between the data for timing the automatic multi-image import versus manual import.

## Results

### New functionality

Significant new functionality has been added to the CPW: allowing the importation of entire image collections into a bench; supporting a user-defined ordering within an image collection; implementing support for long-running tasks, so that they run in the background without adversely affecting the overall system; allowing “Public” benches, so a login is no longer mandatory; adding support for local uploading of miscellaneous images; refining and expanding available search facilities, with the enabling of the ability to tag images for ease of search; and implementing various technical improvements to the system to improve its efficiency, speed, and user-friendliness.

#### Collection import

**Fig. 2:**
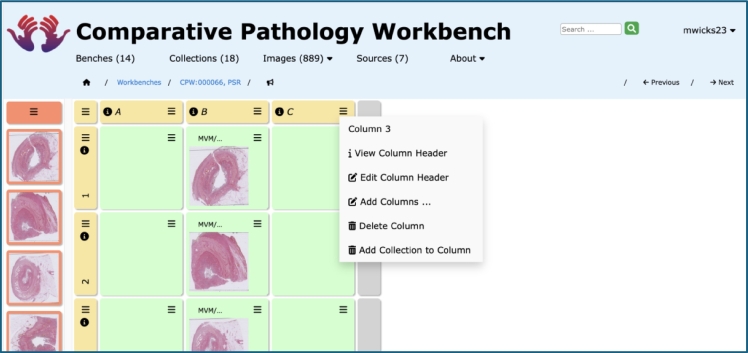
Column header menu item offering collection import. This figure shows the bench page with a bench on the right-hand side of the screen, containing green “ordinary” cells, with row and column header cells in yellow, with a Collection to the left-hand side of the screen. The final menu item for third Column, “3”, “Add Collection to Column” allows the user to populate the column with entire contents of the Collection (as displayed on the left-hand side of the screen). This collection addition has already been done for column “B”. Collection importation is also available for a row; additionally, collection importation can be started from a specific cell, filling the cells to that cell's right or below.

Entire collections of images can now be imported directly into a bench, providing a faster means of populating a bench with images. Previously, a bench could only be updated one image at a time from a collection on the bench page, which can be an onerous task for more than just a small number of images. Typically, a user can create a collection of many tens or hundreds of images from potentially many sources and may need to create a bench containing all these images. The CPW now offers the ability for that user to add all of these images into a single column or row, in a single operation. After importing the entire collection of images, the user is then free to rearrange the bench to their specific requirements. Instead of laboriously populating the bench one image at a time, the user can now import a large number of images in a single operation and spend their time arranging the bench as they require (see Fig. 2).

#### Image sorting within collections

Images can now be sorted within a collection by a user, with collections that are permitted by the collection owner to be viewed or edited by permitted users, having differing sort orders for those users, if required. Previously, there was no order imposed on a collection other than the order in which the images were added to the collection. However, with the possibility of adding an entire collection to a bench, the ordering within a collection became important to the user, as the image order within a collection would be used to populate cells within a row or column. The users required a means to control the image order within the collection, and hence the way images would populate cells down a column or along a row on collection import. The Collection List page has therefore been updated to provide a new menu option, “Edit Image Ordering”, allowing the user via a pop-up, to assign an ordinal number for that image within the total number of images in that collection. Collection image listing can also be sorted by this new user-defined ordering, as well as all the previous sort parameters, such as image “Name”, “Source”, etc.

#### Long-running tasks

**Fig. 3:**
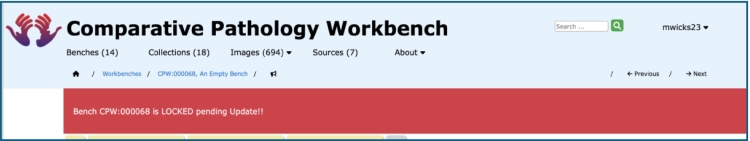
A Locked Bench during a long running update. This screenshot shows a bench that is “Locked” as it is undergoing an update. Typically, this update is a long-running task, so the user needs to be told that no further updates are possible until this update has finished. When the task is finished the bench is “Unlocked” with this banner removed, and then the user is allowed to make further amendments to the bench as required.

Certain tasks within the CPW, e.g., populating a bench with the contents of an entire collection and creating a collection from an entire OMERO dataset, take a long time and previously could cause the CPW system to timeout. The CPW has now been configured to perform these tasks in the “background”, freeing up the user to do other tasks within the system, and avoiding system timeouts. Collections and benches that are being updated this way, are “Locked” until the update has finished (see Fig. 3). When tasks are executed in the background, affected benches and collections are “Locked” while the update is being performed, with a red warning banner heading displayed while the update is being performed. If a user attempts to access the bench or collection while it is being updated, no update options are offered to the user, with only view access possible.

##### A task performance comparison

**Fig. 4:**
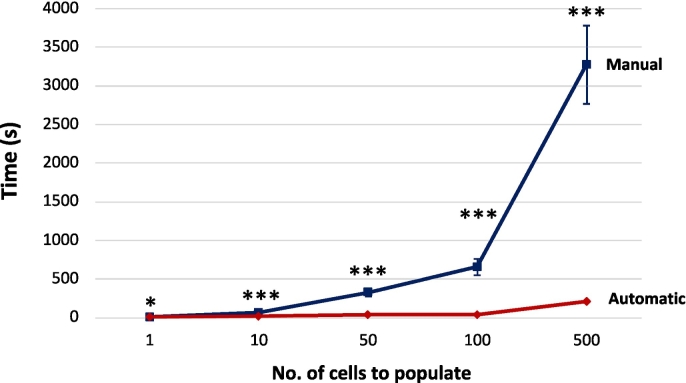
A comparison of manual and automatic bench population with images. Graph showing the relative performance of manual population of a bench with an image one at a time (in dark blue), compared with automatic population with the entire contents of a collection containing 1, 10, 50, 100, and 500 images, respectively (in red). Statistical tests: Welch's *t*-test for summary statistics: *p* > 0.05 ns, *p* ≤ 0.05 *, *p* ≤ 0.01 **, *p* < 0.001 ***, *p* < 0.0001 ****.

A comparison of the relative performance of manual and automatic tasks was carried out, to justify the significant system updates made to the CPW to process tasks in the background outside the web server. Fig. 4 shows the plot of the timings between manual and automatic population of a bench with specific numbers of cells to populate: 1, 10, 50, 100, and 500 images. It can clearly be seen that providing an automatic bench population method offers a significant saving in time for the user, over manual bench population. Note also that the automatic bench population was performed into benches that were sized to already contain sufficient cells to accommodate all images in the entire supplying collection; also note that the extrapolated values for the manual bench population do not include any estimate of the time taken to navigate to the target cell to be populated with an image, suggesting that manual bench population would in fact be considerably longer, in reality. In addition, the automatic bench population does not require any user action, so the user is released to undertake other activities further reducing the time necessary to spend on populating the bench.

#### Public benches

In the first release of the CPW,^11^ all benches, collections, and images were confidential, and required all users to be allocated login credentials and granted access to benches and collections they did not own. It is now possible for selected benches to be marked as “Public”, and as a result these benches can be viewed without the user having specific access rights. Whereas it is now possible for the unauthenticated user to view benches that have been marked as “Public”, it is only possible for that type of user to view any images contained in the bench as thumbnail images; if the image referenced in a cell of a bench is hosted by a system that requires further authentication credentials, then those credentials are still required and access is denied to the unauthenticated user of that image hosting system. Public benches can be accessed by clicking on the new card on the CPW Home page, where a list of public benches will be displayed.

#### Miscellaneous images

**Fig. 5:**
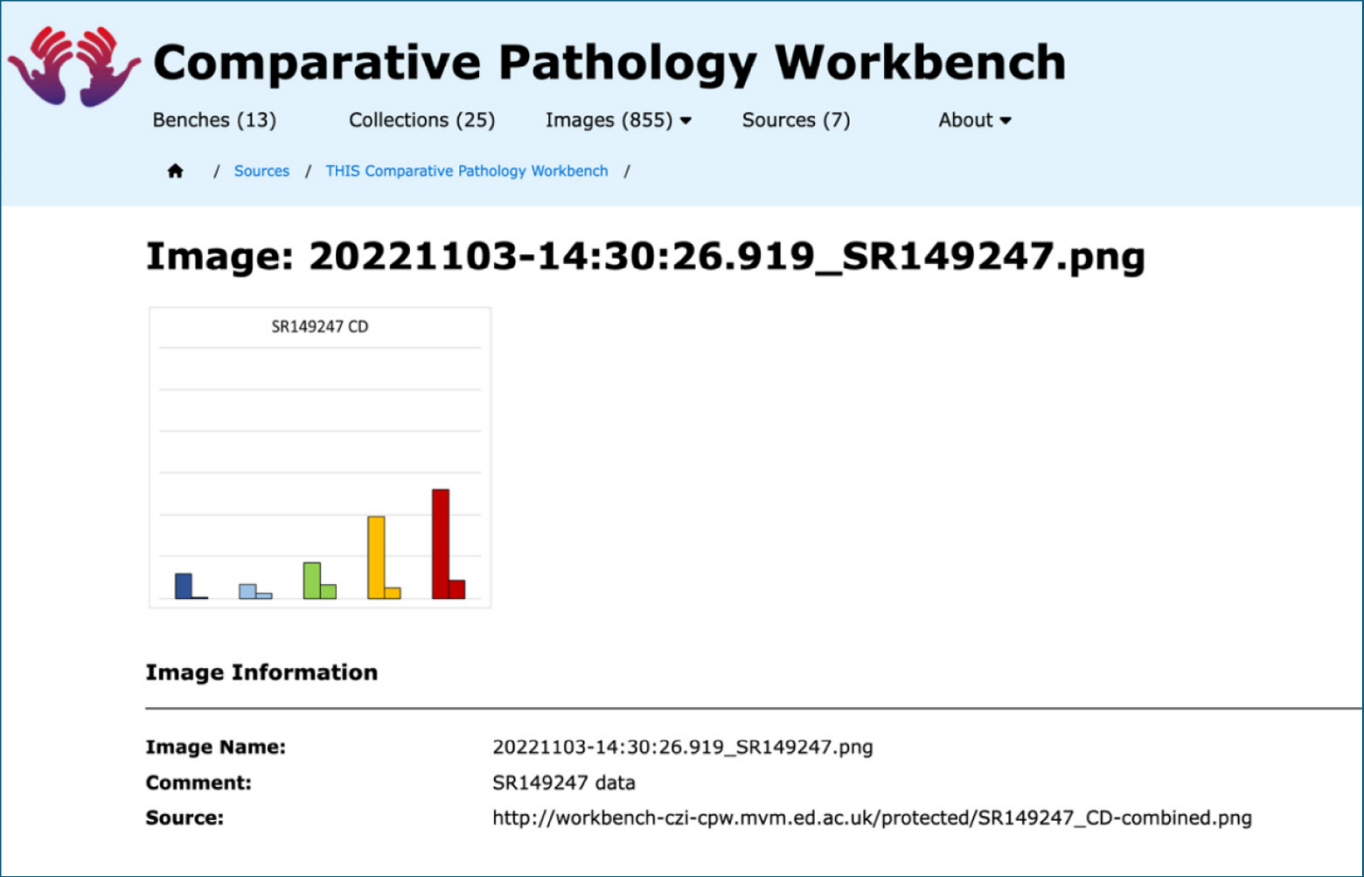
An example miscellaneous Image uploaded directly to the CPW. This screenshot shows the Image Information page for an image uploaded directly to the CPW. Sometimes, the user may want to incorporate miscellaneous images on a bench, e.g., containing the result of some analysis performed on other images held in the same bench. In this case, this image is a chart generated by and exported from Microsoft Excel, as a PNG image. It would also be possible to upload such an image to an available OMERO server, but not an appropriate use of sophisticated image storage resources. It is possible to upload such miscellaneous images to the WordPress server associated with the instance of the CPW, but this relies on this mechanism being available on the WordPress server, which may not be the case for all implementations of the CPW. Instead, the CPW has been augmented with the ability to store such miscellaneous images locally in the system, as shown above.

The CPW can support several types of image source, including OMERO servers, WordPress servers, and data from the EBI SCEA^2^^,^^5^; images from these sources are then displayed as references in a bench, with thumbnail images to represent the external images hosted elsewhere. In addition, the CPW can now support the use of miscellaneous images, which can then be displayed in a bench, as shown in Fig. 5.

#### Search Options

The available Search Options within the CPW have been considerably enhanced and expanded, with the addition of a Simple Image Search, in addition to the existing full Image Search, which has been renamed the Advanced Image Search. To aid the Advanced Search, Tagging of Images has been introduced, where user-defined text strings can be associated with required images, allowing the user a means to directly find specific images with that tag, producing a faster search result. These user-defined tags can also be used in combination with all other existing advanced search options. The Global Search option has been refined to return the first 10 instances of an image, collection, and bench, where the name matches the supplied string.

##### Global Search

**Fig. 6:**
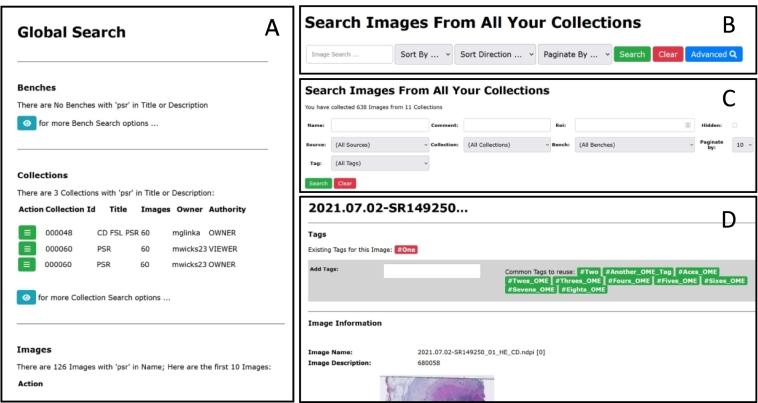
New search options. A: Global Search is initiated from the search box in the page header or from the “Global Search” page. Entering text into the box and hitting return produces a summary of the first 10 Benches, 10 Collections, and 10 Images that contain the supplied string in this case “psr”. B: With “Simple” Image Search, the user can enter a search string, with a selection of sort criteria, being field and direction, and the required pagination. In the returned data, the green “hamburger” menu icons, together with the blue-gray “eye” icons, allow the users to access further search options or further Bench, Collection, and Image details. The search results can be sorted by any attribute and direction, using the green buttons, and paginated as required. C: The Advanced Image Search page allows detailed search of the “Name”, “Comment”, and “Roi” fields. Selecting “Hidden” allows hidden images to be included in the search. The fields “Source”, “Collection”, “Bench”, and “Tag” defining specific constraints for each category. Combinations of these parameters refine the required search. D: Tags (text strings) can be allocated to an image. Tags already allocated to the image are shown in red, other tags available in the system are shown in green. These unallocated tags can be clicked on to associate that tag with this image. Otherwise, a text box is provided where the user can type in a new tag string to allocate a new tag to this image. Once tags have been allocated to an image, they can be used in subsequent pages in the system, e.g., searching by Tag in the Advanced Search page.

The Global Search can be accessed anywhere in the CPW system, using the simple text search box in the main header panel. This text box which takes the supplied (case sensitive) text string and returns a list of benches, collections, or images, whose title or description contains the supplied string, to which the current user has access, as illustrated in Fig. 6A.

Detailed additional search options are offered on the search results page of the Global Search, as shown in Fig. 6A.

##### Simple Image Search

The new Simple Image Search is available from the Images option on the CPW Main Menu. This provides a simpler means to search for images than the existing search facility, which has been renamed the Advanced Image Search. In the Simple Image Search, the supplied text string from the text box is used to search for the image by their names only, with the results sorted using the selected field and selected direction, from the matching dropdown menu options, with the results paginated according to the selected pagination dropdown menu. Fig. 6B shows the Simple Image Search page.

##### Image tags

The CPW has been modified to support the adding of tags to images; these tags can be used to search for specific subsets of images in collections or benches. Images can have tags added from the set of existing tags, or a completely new tag can be added. The CPW can separately support tags associated with images stored on OMERO servers, however, there is no support for the adding or editing of this type of tag within the CPW. Once a tag has been added to an image, it can be used in the Advanced Image Search to more easily find images (see Fig. 6C), as well as being used to sub-select tagged images from the selected collection on the Bench page.

Fig. 6D shows the Tag Image page, accessible from the Advanced Image Search Results pages, where a menu option is offered to allow a user to Tag an image. The Tag Image page allows the user to add or remove existing Tags, or add a completely new Tag, if required.

##### Advanced Image Search

The Advanced Image Search is also available from the Images option on the CPW Main Menu. This page provides a more comprehensive means to search all the images available to the user, using the Name, Comment, ROI, Hidden, Source, Collection, Bench, and Tag, and their combinations. The search results can be sorted by any attribute and direction, together with the desired pagination amount, as shown in Fig. 6B and C.

#### Other additional functionality

The final updates to the CPW are mostly technical in nature, with an update to the existing REST interface, and the implementation of local caching of image thumbnails. Whenever an image reference is added to a collection from an OMERO server, e.g., a thumbnail image is generated, stored locally, and used throughout the system when that image reference is displayed. This avoids the constant rendering of the image reference, which generates considerable online traffic, and can make the display of webpages in the CPW very slow, particularly when a bench contains many image references. Finally, all locally cached thumbnails are securely stored on the web server, and access is only granted if the request to view the thumbnails is made by an authenticated user of the CPW.

#### Software upgrades

The remaining updates to the CPW have been environment upgrades—the CPW can now run within the Conda virtual environment manager. Other virtual environments are available, and there is no reason why the CPW could not run within them, but they have not been tested. The CPW has been upgraded to run with Python 3.8 and Django 4 with all the required Python Packages upgraded to match these versions. Version 2.0 release of the CPW on Github^11^ requires these versions of Python and Django and is compatible with PostgreSQL 15.

### Bench exemplars

Here, we illustrate usage of the CPW via four examples of “real-world” applications. Three examples are part of clinical pathological research programs and one example is teaching-based.

#### Updated bench exemplars

In this section, we provide updated or new CPW exemplar benches that used CPW v2.0.

##### DERMATLAS expert diagnosis review

The DERMATLAS CPW bench was updated using CPW v2.0 to use automated uploading of additional very large Collection image datasets, with pre-upload ordering and more efficient collaborative diagnostic expert user analysis of uncommon and rare skin tumors to reach consensus diagnosis amongst a set of experts for use in genomic sequencing of several cohorts of skin tumors. This took advantage of the important update of the CPW that involved working with large datasets and the difficulties encountered in the past with them. This included uploading and sorting large collections, without the system being timed out. Furthermore, the significant work of making a large bench is now rewarded twice, with the possibility to make it publicly available when publishing the article (which is increasingly being requested by journals), in addition to the work required for the article itself. Finally, the improvement of the search facility has proven very helpful, when working with many different skin tumor histopathological image datasets. Although the search facility is case sensitive, the option of using an asterisk in the search string as a “wild card” was particularly useful in searching the DERMATLAS CPW benches for selected tumor cases for additional expert review. For example, using the following search string in the image search “XY-18*Z1*2021-02-09*” returns 2 images: “XY-18-70754 Z1 - 2021-02-09 14.41.56.ndpi” and “XY-18 14411 Z1 - 2021-02-09 14.32.36.ndpi”.

##### Coeliac disease duodenal biopsy AI diagnosis

In a recent study evaluating AI-based diagnosis of celiac disease on scanned duodenal biopsy histopathological section images,^14^ we used the updated CPW v2.0 to facilitate image review and diagnostic comparison. A dedicated CPW workbench was created containing 30 biopsy section images: 10 from cases previously diagnosed as normal, 10 diagnosed as coeliac disease, and 10 with other diagnoses (non-normal and non-celiac, such as adenoma, non-specific inflammation, giardiasis, etc), arranged in a single column. Each image linked to the corresponding whole slide image was hosted on an OMERO instance. Four pathologists accessed the workbench remotely and simultaneously, using the interactive interface to review the images and record their diagnoses. These independent diagnoses were then used to assess pairwise agreement between pathologists and to compare each pathologist's classification with that of the AI system. The new updates simplified the setting up process of the workbench and the user-friendliness of its operation by expert pathologists.

##### Crohn's disease fibrostenosing lesion components analysis by ileal layer

Version 1.0 of CPW was previously used to create a workbench in order to discuss the changes present in whole slide images of full ileal wall sections of Crohn's disease fibrostenosing lesions. In the previous work by Wicks et al.,^6^ the functionalities present in the workbench allowed for a discussion and commentary on the status of the tissue samples. This project provided feedback pinpointing the necessity to expand the tissue analysis for the various layers within the ileal wall, including the markedly expanded muscularis mucosae layer. Furthermore, the ability to add miscellaneous data charts to the CPW v2.0 bench associated with each of the samples being analysed by immunohistochemistry for a range of cell types, helped to provide a much more informative investigation.^21^

The recent updates to the functionalities of CPW v2.0 allowed for generation of a 540-cell bench (9 columns and 60 rows) that was created in order to provide public access to the images used in the Crohn's disease research by Glinka et al.^21^ on the normal ileal wall controls and Crohn's disease fibrostenotic lesion ileal wall full thickness histopathological sections. The images contained within the cells linked to the public OMERO instance, allowed sharing and visualizing of individual cases that were hand-annotated in QuPath.^9^ The CPW version 2.0 ability to automatically populate whole columns from pre-sorted collections allowed for rapid generation of a publicly available bench for visualization of data used. The new functionalities allowed for more convenient expansion of the previous use case in order to show all of the samples involved in the study, and not just a few example sections.^22^ With the ability to visualize, all ileal wall layer samples together the relevant stains, including the histochemical stain PicroSirius Red for collagen deposition analysis and a wide range of immunohistochemical stains used to identify and quantify specific cell types, this new and considerably upgraded bench allowed for much enhanced discussion between expert histopathologists resulting in formation of a new hypothesis for the pathogenesis of Crohn's fibrostenosis as described by Glinka et al.^21^ that emphasized the importance of the interaction between Crohn's lymphoid aggregates and surrounding endothelial cells in the disease mechanisms. With the ability to easily and rapidly generate large workbenches with pre-ordered images, the CPW v2.0 provides an invaluable tool for consecutive images being placed one next to another for both analysis and generation of atlases.

##### A veterinary pathology teaching image bench

The CPW v2.0 is able to support teaching of undergraduate veterinary students in both the topics of histology and histopathology in veterinary schools. This is achieved by providing structured access to a curated database of annotated gross and histological images, organized by organ system, disease process, and etiology. Its image-enabled “spreadsheet” layout allows students to compare cases and stains visually (e.g., compare hematoxylin and eosin-stained sections with sections stained with Masson's trichrome for the detection of fibrosis). Concurrently, tagging, search, and annotation features enable targeted questioning and interactive learning. For example, it is possible to create test questions on specific histological pattern in a slide's discussion thread, that relate to specific annotations of that pattern in the slide image. This test question delivery method can be used also as part of formative or summative assessment.

Similarly, for professional training (e.g., continued professional development courses and professional conferences), CPW facilitates the private publication of curated slide sets via public benches. Educators can share annotated images with text and image annotations, to generate high-quality reference material. Refined search tools and an improved interface support efficient navigation and learner engagement during courses or self-directed study. An additional advantage is the possibility of using the discussion thread to capture questions, which can be addressed by the educator within the system, so that all attendees can view the answer.

Finally, the CPW also enables collaborative veterinary pathology research and commercial consultancy through secure, private sharing of annotated datasets. This is especially useful in comparative pathology services that produce pathological data for research projects or commercial projects (e.g., toxicopathology assessment, medical device assessment studies). Users can review and edit benches collaboratively, with discussion threads supporting remote peer review and consensus interpretation. New features such as long-running task support and improved collection management streamline work with large, complex datasets in veterinary pathology.

## Discussion

The CPW^6^ (version 1) has already supported a number of research programs. ^6^^,^^13^^,^^14^ In discussion both with the existing users and new projects, we identified shortcomings and additional capabilities. In this manuscript, we have set out how these issues have been addressed and shown, via exemplar studies, how CPW version 2 delivers the required functionalities.

The existing method of importing images into a bench was laborious as only one image at a time could be imported. The importation of multiple images from both an image source into a collection, and from a collection into a bench, was too slow, and sometimes caused the system to timeout with a consequential loss of user-effort. Sometimes users needed to import images from sources other than image servers into benches. Whereas benches and collections could be shared between CPW users, users wanted to be able to share benches with unregistered CPW users. Users found the existing search facilities too complicated. Users also found that creating large collections of images was easy to achieve, yet these large collections were unwieldly to use, and hard to find images within large collections. Finally, various technical enhancements to the CPW were required to improve the efficiency, speed, and ease of use of the system.

To address these issues, we implemented means to import an entire collection into a bench, by populating a specific column or row of cells with images, as a new function in the header menu or cell menu. This uses background processing via the Redis^20^ message broker and Celery^19^ task scheduling software; this leaves the user free to continue using the CPW for other tasks, while the background tasks are completed. Further to this new image import functionality, we have implemented a supporting collection ordering function to enable the users to determine the order in which images populate cells, when importing an entire image collection, with a new cell labeling system, thus making the process of bench population with images much more efficient.

We have implemented a mechanism to upload miscellaneous images to the CPW itself, thereby allowing charts from Microsoft Excel and other commonly used programs, to be imported into benches. We have created “Public” benches, which can be viewed by unregistered CPW users. We have updated significantly the available search facilities in the CPW: there is the Global Search in the Header panel, which gives a “snapshot” of any matching images, collections, and benches; there is a new Simple Image Search for images, providing a text search on image name with minimal sort parameters; finally, the existing image search remains as the Advanced Image Search for complex combinations of search parameters. Whereas the users can now create very large collections, finding a particular image or set of images within a collection was previously time-consuming, so we have introduced the ability to tag images, and to recognize and use tags that may have been used on the external image servers where they reside. Tags can be used in two main ways: within the Advanced Image Search as an extra means of finding an image or subset of images; or within the Bench page to sub-select a large collection that is displayed at the same time. Additionally, as other software has evolved, the packages and systems that the CPW uses have either been upgraded, are no longer supported, or required consequential upgrades to continue using the features we require; the CPW version 2 release on Github that accompanies this article,^23^ now supports Python 3.8, Django 4, and PostgreSQL 15.

As the improvements have been implemented from version 1 to 2 of the CPW, there are limitations within the system that may need to be addressed in the future, particularly when the user-base increases. The “locking” mechanism, as described earlier, blocks the access to the bench for all users once a user automatically populates a bench. Edge-cases where two users simultaneously access and perform similar or the same actions will have to be addressed. The cell labeling by grid coordinates provides users with the ability to easily locate the cell of interest, but further enhancement of the system would be helpful, e.g., where user-changed titles for the columns and rows could be reflected by the cell coordinate system. As mentioned earlier, additional sources as well as OMERO have been implemented; with more sources, such as those involving spatial transcriptomics, are being examined to further enhance the functionality of CPW.

## Conclusions

In an earlier article,^6^ we described version 1 of the CPW, here we present an upgraded version 2 of the CPW, driven by collected user experiences that have coalesced around seven specific user requirements. We have described resolutions to these user requirements, as well as further technical enhancements to the software infrastructure. We have backed up these user driven requirements, by reviewing the user experiences of three previous exemplar use cases (DERMATLAS expert diagnosis review of uncommon and rare skin tumors),^1^ a Crohn's disease fibrostenosing lesion comparative analysis, and a celiac disease duodenal biopsy section AI diagnosis project, and improving their utility and efficacy with two further exemplar use cases (a further Crohn's disease comparative analysis, and a veterinary pathology teaching image system). Thus, this new CPW version 2 has already been “road tested” within the human and veterinary pathology communities and found to be a significant improvement. The CPW can be applied to any area of research or teaching or development that focusses on visual image comparison and community analysis, and we look forward to supporting many different applications in other fields.

## CRediT authorship contribution statement

All – manuscript preparation and review;

MNW - primary software development and implementation;

MG, IF, SD, KK – application testing and review, bench applications;

BH, DH, MS – software review and application testing;

DA, IP - PI support and data sources;

AB – PI support and Comp. Sci. input;

RAB, MJA – PI support, original design concepts, applications testing and bench development.

## Ethics approval and consent to participate

Not applicable.

## Funding

Chan-Zuckerberg Initiative Human Cell Atlas grant A-1708-02723, The Leona M. and Harry B. Helmsley Charitable Trust entitled “Human Gut Cell Atlas – Normal Intestine and Crohn's Disease”, grant reference number 1903–03783 and the Medical Research Council grant MR/V000292/1 entitled “The Genomic Atlas of Dermatological Tumours (DERMATLAS)”.

SD acknowledges funding from NHS Lothian RD.

## Declaration of competing interest

The authors declare the following financial interests/personal relationships which may be considered as potential competing interests:

Mark J Arends and other co-authors report research financial support was provided by Chan-Zuckerberg Initiative Human Cell Atlas grant A-1708-02723, entitled “Comparative Workbench for Atlas Data”.

Mark J Arends and other co-authors report research financial support was provided by The Leona M. and Harry B. Helmsley Charitable Trust, grant reference number 1903–03783, entitled “Human Gut Cell Atlas – Normal Intestine and Crohn's Disease”.

Mark J Arends and David J Adams report research financial support for skin tumor research was provided by 10.13039/501100000265Medical Research Council, grant MR/V000292/1 entitled “The Genomic Atlas of Dermatological Tumours (DERMATLAS)”.

Mark J Arends reports research financial support for coeliac disease research by both an 10.13039/501100006041Innovate UK/Coeliac UK grant entitled “Development of an artificial intelligence solution for diagnosis and assessment of severity of pathology in small intestinal biopsies in suspected or known coeliac disease” and a 10.13039/501100009270NIHR Invention for Innovation (i4i) Call 25 A Stage 2 grant entitled “Optimisation and clinical validation of a digital diagnostic system for small bowel biopsies” (NIHR205502).

Shahida Din reports financial support was provided by NHS Lothian Research and Development.

The other authors declare that they have no known competing financial interests or personal relationships that could have appeared to influence the work reported in this article.

## Data Availability

All software developed for this project is freely available from the GitHub repository: https://github.com/Comparative-Pathology/comparativepathologyworkbench/releases/tag/Release-Candidate-2.0.0
